# Two novel homozygous mutations in *NPHP1* lead to late onset end-stage renal disease: a case report of an adult nephronophthisis in a Chinese intermarriage family

**DOI:** 10.1186/s12882-019-1372-4

**Published:** 2019-05-16

**Authors:** Yiting Wang, Feng Chen, Jiali Wang, Yingwang Zhao, Fang Liu

**Affiliations:** Division of Nephrology, West China Hospital of Sichuan University, No. 37, Guoxue Alley, Chengdu, 610041 Sichuan Province China

**Keywords:** Nephronophthisis, Late onset, End-stage renal disease, Whole-exome sequencing, Genotype, Phenotype

## Abstract

**Background:**

Nephronophthisis (NPHP) is an autosomal recessive hereditary disease with highly variable clinical characteristics for which 20 genes (*NPHP1–20*) have been identified. NPHP1 is the major subtype leading to pediatric end-stage renal disease (ESRD). Reports of adult NPHP1 are rare.

**Case presentation:**

Here, we report a 27-year-old male from a Chinese intermarriage family who was diagnosed as NPHP from clinical presentations and molecular genetic analysis by whole-exome sequencing. The genetic investigation revealed a novel homozygous nonsense mutation, p. E697X,37 and a novel homozygous missense mutation, p. F691 L, in the *NPHP1* gene. His parents and fraternal twin harbored heterozygous mutations of the two loci and had no renal symptoms. His elder sister developed ESRD and died at 23 years of age.

**Conclusions:**

The report indicated that adult NPHP should be taken into consideration for adults with ESRD of uncertain cause. The genotype-phenotype correlation requires further investigation.

## Background

Nephronophthisis (NPHP), an autosomal recessive hereditary disease, is a major cause of pediatric end-stage renal disease (ESRD) with an estimated incidence of 1:50,000 in Canada and 1:1,000,000 in the United States [1–4]. The clinical characteristics are highly variable and causal mutations in 20 genes (*NPHP1–20*) have been identified (https://omim.org/search/?index=entry&sort=score+desc%2C+prefix_sort+desc&start=1&limit=10&search=%3DNephronophthisis). However, the genotypes of approximately 60% of NPHP patients remain unclassified [5]. Homozygous deletion mutations in N*PHP1* are the most common genotype causing NPHP1 [3, 5, 6]. The clinical characteristics of NPHP1 are generally nonspecific and mostly limited to the kidney, which commonly presents with polydipsia, polyuria, secondary enuresis and renal dysfunction. In some cases, the liver, pancreas, visual system and central nervous system (molar tooth sign on brain Magnetic Resonance Imaging) are involved [7]. A study from Egypt found that among 20 NPHP1 patients (the mean age at diagnosis was 87 months), 95% had typical NPHP symptoms of polydipsia, polyuria and secondary enuresis and 75% of them presented with sign of ESRD [8].

Almost all the patients with deletion-causing NPHP1 develop ESRD by the age of 19 years [7, 9, 10]. Adult NPHP1 with late onset ESRD can easily be ignored by nephrologists in clinical practice. Recently, single-nucleotide polymorphism genotype identified 26 patients with homozygous *NPHP1* deletions among 5606 European patients with adult-onset ESRD. Before this genotyping, only three (12%) of the 26 patients were classified as NPHP. One of the 26 had proteinuria, but polyuria and ophthalmological and neurological anomalies were not detected. The others had been misdiagnosed with other nephropathy (46%) or chronic kidney disease of unknown etiology (42%) [11]. The clinical heterogeneity of NPHP, particularly in adults, highlights the important role of genetic identification. However, diagnosis based on clinical presentation or Sanger sequencing alone appears to be inaccurate and time-consuming. In this study, we report two novel mutations in the *NPHP1* gene detected by whole-exome sequencing that cause adult NPHP1 with late onset ESRD in a Chinese intermarriage family.

## Case presentation

### Clinical report

**Fig. 1:**
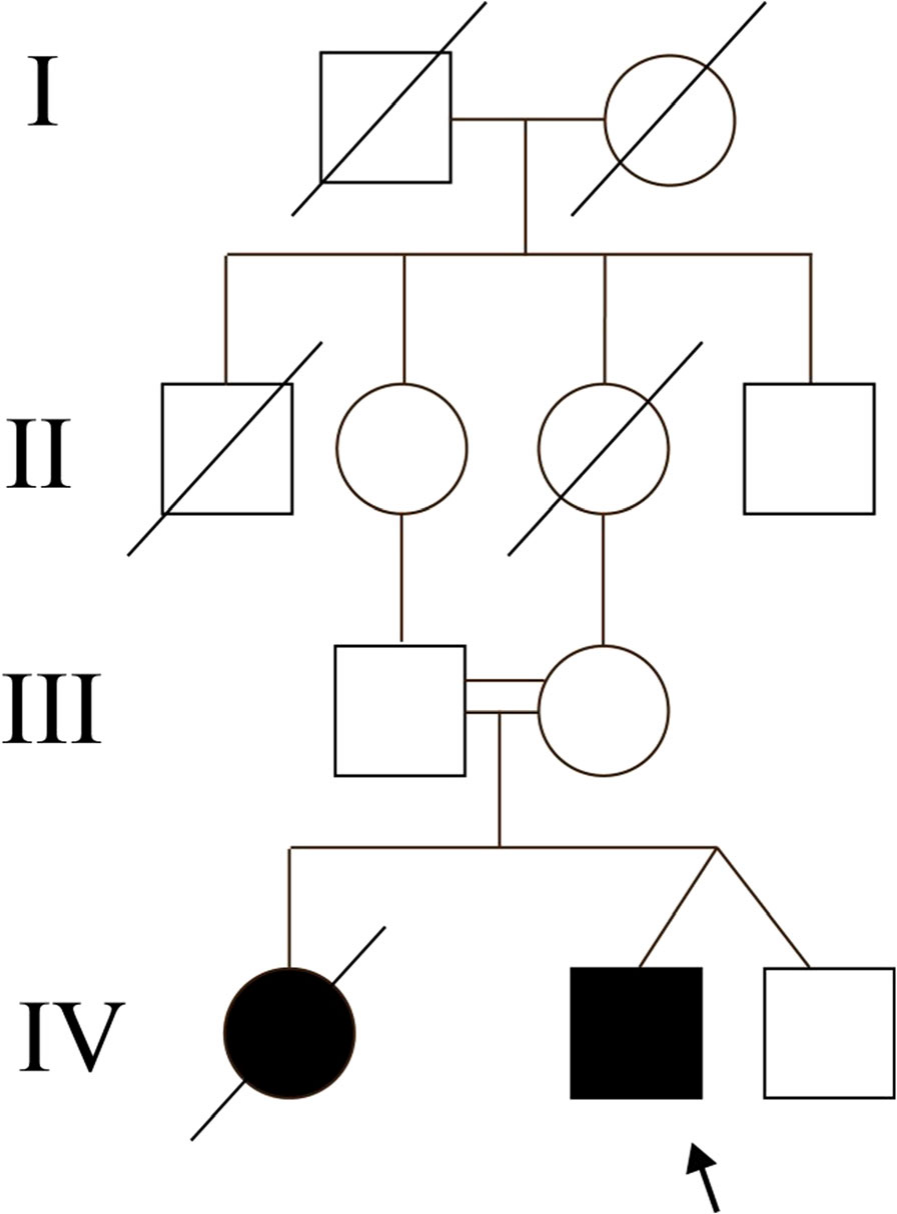
Family tree of the proband. represents proband, represents male, represents female, represents intermarriage, represents fraternal twin, represents dead; represents patients with renal phenotype. Proband harbors homozygosis mutation of p. E697X,37 and p. F691 L in *NPHP1* gene. Parents of the proband are first cousins and they harbor the two heterozygous mutations

A 27-year-old Chinese male was defined as the proband, and his family members were investigated. The chief complaint of the proband was feeling weak for 6 days. His parents were first cousins and both of them were healthy. The proband’s fraternal twin did not display any biochemistry or imaging abnormalities. Six years previously, the proband’s elder sister died due to kidney failure at 23 years of age. The family tree is depicted in Fig. 1.

The clinical, biological and radiological features of the proband and his healthy fraternal twin are presented in Table 1. Briefly, the height of the proband (159 cm) was shorter than that of the average adult male. Laboratory investigations revealed significant elevations in the levels of serum creatinine (420.0 μmol/L), cystatin-C (2.83 mg/L) and parathyroid hormone (83.38 pmol/L). The estimated glomerular filtration rate was 14.84 ml/min.1.73m^2^. Ultrasound scans showed the size of right kidney size to be 9.1 × 4.3 × 4.2 cm and that of the left kidney size to be 9.0 × 3.7 × 3.3 cm. Both kidneys were of nearly normal size and each had a cortical microcyst (less than 1.5 cm) respectively. Blood pressure, urinalysis, serum liver enzyme levels, lipid metabolism makers, serum uric acid level, electrolytes and immune system makers were normal. Computed tomography scans revealed normal abdominal and cerebral structures. Renal biopsy was not performed because of deteriorated kidney function. The proband denied any medical history involving urinary, visual or auditory systems, including polydipsia, polyuria and secondary enuresis. After follow up for one year with treatments including medicinal charcoal tablets and rocaltrol, the serum creatinine level was 414.0 μmol/L, the estimated glomerular filtration rate was 15.77 ml/min.1.73m^2^ and the parathyroid hormone level was 19.21 pmol/L. According to the clinical presentations and family history, genetic kidney disease was suspected and whole-exome sequencing was performed on close family members to make a molecular diagnosis.

**Table 1:** Clinical, biological and radiological features of proband and his unaffected brother

	Proband (male)	Unaffected brother
Age (years)	27	27
Height (cm)	159	161
BMI (kg/m^2^)	23.4	24.0
Blood pressure (mmHg)	124/80	116/82
Age of onset	27	–
Onset of ESRD	–	–
Hemoglobin (g/L)	131	
Albumin (g/L)	47.7	51.2
Serum creatinine level (μmol/l)	420.0	84
Evaluated glomerular filtration rate (ml/min per 1.73m^2^)	14.84	109.32
Cystatin-C (mg/L)	2.83	0.88
Uric acid (μmol/L)	489.0	335.0
Parathyroid Hormone (pmol/L)	83.38	4.46
Polyuropolydipsia	negative	negative
Haematuria	negative	negative
Proteinuria (g/L)	0.7	negative
Kidneys size (cm)	RK: 9.1 × 4.3 × 4.2 LK: 9.0 × 3.7 × 3.3	RK: 9.7 × 4.6 × 4.5 LK: 9.6 × 4.0 × 3.6
Cysts (cm)	RK: 1.5 × 1.4 LK: 0.6 × 0.5	none
Abdominal CT scan	Normal	–
Cerebral CT scan	Normal	–
Neurological development delay	Normal	–

“-“represents the examination did not perform. “RK” represents right kidney,” LK” represents left kidney

The study protocol was conducted based on the principles of the Declaration of Helsinki. Written informed consent was obtained from all participants.

### Genetic investigation-method

Peripheral blood samples of the proband and close family members (his parents and fraternal brother) were collected and whole-exome sequencing was performed according to standard protocols. Briefly, samples were extracted using a Qiagen genomic DNA isolation kit (Qiagen, Hilden, Germany); genomic DNA samples were sheared by sonication. The sheared genomic DNA was then hybridized with NimbleGen 2.0 probe sequence capture array Roche, (http://www.nimblegen.com/products/seqcap/ez/v2/index.html) to enrich the exonic DNA (Joy Orient, China). The libraries were first tested for enrichment by qPCR and for size distribution and concentration using an Agilent Bioanalyzer 2100. The samples were then sequenced on an Illumina Hiseq2500. Two parallel reactions were done for each sample.

### Genetic investigation-results

**Fig. 2:**
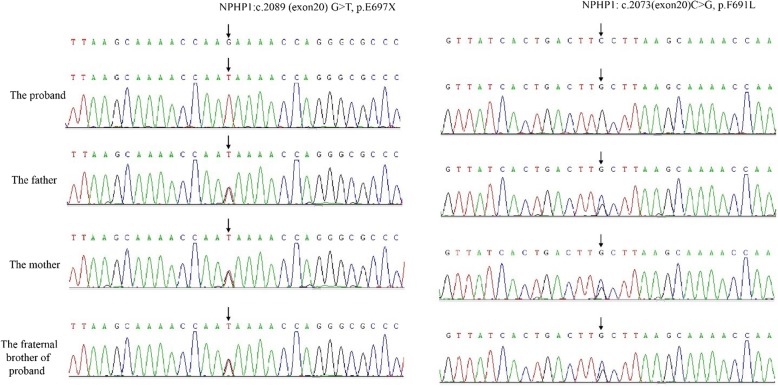
Sanger sequencing of the two mutations

A novel homozygous mutation, c.2089 G > T in exon 20 of *NPHP1* was identified in the proband, resulting in a nonsense alteration of p.E697X,37, which led to a truncation of 37 amino acids. Both the proband’s parents and his fraternal brother were heterozygous for this mutation. All the variants were verified by Sanger sequencing (Fig. 2). The mutation was not reported previously in the public domain single-nucleotide polymorphism databases: dbSNP (http://www.ncbi.nlm.nih.gov), ExAC (The Exome Aggregation Consortium; http://exac.broadinstitute.org), and control exome sequencing data of 1000 ethnic Han. Moreover, another novel homozygosis mutation c.2073 C > G in exon 20 of *NPHP1* was detected, resulting in p.F691 L, which was predicted to be deleterious by the Sorting Intolerant from Tolerant algorithm (SIFT; http://sift.bii.a-star.edu.sg/). The proband’s parents and his fraternal brother were also heterozygous for this mutation.

## Discussion and Conclusions

NPHP is a genetic disorder with highly variable clinical presentations. The majority of children with NPHP develop ESRD between the ages 11 to 13 years [7]. Genetic identification is the golden standard for accurate diagnosis. Compared with Sanger sequencing, whole-exome sequencing is more time saving, effective and available along with the improved technique and reduced price. In this study, a novel homozygous nonsense mutation, p.E697X,37, and a novel homozygous missense mutation, p.F691 L, were identified in *NPHP1* by whole-exome sequencing in an adult with late onset ESRD. The proband’s parents and fraternal twin harbored were heterozygous for both loci without any clinical abnormality. As we know, interaction between mutations at different loci in a gene may result in different clinical phenotypes. The homozygous nonsense mutation caused a truncation of the protein is likely to be decisive in causing the disease, while the other missense mutation may modify its effect [12–14]. The cumulative effect of the two *NPHP1* mutations might allow us to analyze the genotype-phenotype correlation.

Based on the age of ESRD onset, NPHP1 can be classified into three clinical forms, infantile, juvenile and adolescent. The median age of onset for juvenile ESRD is about 13 years old, and that for the adolescent form is younger than 19 years of age [5]. The oldest age of onset for renal symptoms in a recent report of 60 NPHP1 patients was 16 years old [7]. Recently, out of 5606 renal transplant recipients in Europe, 26 patients were re-diagnosed with NPHP1. Previously 88% of these 26 patients were classified as other chronic kidney disease. Among them, the median age of initial renal replacement therapy was 30 years old [11]. In the current report, the onset age of renal dysfunction was 27, and his sister died of uremia at 23 years of age. To our knowledge, this is the first report of a case of adult NPHP1 with late onset ESRD in a Chinese intermarriage family [15–17]. The clinical heterogeneity of the renal phenotype onset age may be ascribed to the type and position of the mutations and the interaction between them. The homozygous nonsense mutation caused a truncation of 37 amino acids of each DNA strands. The resulting protein of 696 amino acids may function normally; however, the truncated protein would be vulnerable and may eventually function abnormally. The protein would work normally with the heterozygous mutation.

Nephrocystins, encoded by *NPHP* genes, are located in primary cilia and are essential for ciliary function. Mutations in *NPHP* genes result in ciliopathy and the involvement of different organs leads to different clinical syndromes [18, 19]. Joubert syndrome is diagnosed based on the presence of a molar tooth sign on cerebral magnetic resonance imaging; Senior–Løken syndrome presents NPHP and retinitis pigmentosa; COACH syndrome is characterized as additional hepatic fibrosis and/or ocular coloboma [20]. Only two patients displayed extrarenal anomalies among 26 adult patients with NPHP1 [11]. In a recent report, most of the patients (77%) with NPHP1 presented with isolated renal phenotypes, compared with 34% of patients with other NPHP subtypes. Within the NPHP1 cohort, the majority of patients reached ESRD between 11 to 13 years of age and the progression of chronic kidney disease was much faster [7]; however, the family in this report presented high heterogeneity. Despite the tremendous advances in deciphering the molecular genetics of NPHP, the genotype-phenotype correlation is still beyond our understanding; even within the same family, mutations in the same locus can not fully explain the varied clinical manifestations. The cumulative effect of mutations and the interaction between them need to be considered. Of course, in addition to genetics, the onset and progression of NPHP are influenced by environmental, behavioral, biological risk factors that interact with the genetic background [21, 22].

Sixty percent of pediatric NPHP patients can-not be genetically classified [5, 7] and accurate diagnosis of adult NPHP is much rarer. Therefore, a biobank of large sample size and further in vivo and/or in vitro experiments are imperative. Research into genotype-phenotype correlation with respect to the onset and progression of NPHP, will shed light on the complex pathogenesis of this disease. This will facilitate prevention, benefit early diagnosis and help to tailor intervention to reduce the incidence and minimize progression.

A novel homozygous nonsense mutation, p.E697X,37, and a novel homozygous missense mutation, p.F691 L, in *NPHP1* were identified causing adult NPHP1 with late onset ESRD in a Chinese intermarriage family. Although research into genotype-phenotype correlation is needed, intermarriage should be avoided to prevent autosomal recessive hereditary diseases and breaking the chain of genetic disorder.

## Abbreviations

ESRD: End-stage renal disease
ExAC: The Exome Aggregation Consortium
NPHP: Nephronophthisis
